# Strength Properties of Structural Glulam Elements from Pine (*Pinus sylvestris* L.) Timber Reinforced in the Tensile Zone with Steel and Basalt Rods

**DOI:** 10.3390/ma14102574

**Published:** 2021-05-15

**Authors:** Radosław Mirski, Marcin Kuliński, Dorota Dziurka, Marta Thomas, Ryszard Antonowicz

**Affiliations:** 1 Department of Wood-Based Materials, Poznań University of Life Sciences, ul. Wojska Polskiego 28, 60-627 Poznań, Poland; maricn.kulinski@up.poznan.pl (M.K.); dorota.dziurka@up.poznan.pl (D.D.); 2 Faculty of Civil and Transport Engineering, Poznan University of Technology, Piotrowo 5, 60-965 Poznań, Poland; marta.thomas@put.poznan.pl; 3 Faculty of Civil Engineering, Wrocław University of Science and Technology, Wybrzeże Wyspiańskiego 27, 50-370 Wrocław, Poland; ryszard.antonowicz@pwr.edu.pl

**Keywords:** glulam elements, pine timber, reinforced beams, strength properties

## Abstract

The study analyzed potential applicability for asymmetric reinforcement of glulam beams using materials with a higher modulus of elasticity. Reinforcement elements included smooth and ribbed steel rods as well as basalt rods. These rods were placed only in the tensile zone, assuming that they will not only impart increased rigidity but first of all will reduce the scatter of bending strength values. What is significant, tests were conducted on timber with defects, as it is most commonly used in industrial practice. Analyses showed that this provides an increase in rigidity close to the assumed level. A significant increase in strength was observed. The manufactured beams reinforced with steel and basalt rods were characterized by mean bending strength amounting to 54 and 47 N/mm^2^, respectively. However, no significant improvement was found in the scatter of the observed variable. Beams reinforced with steel exhibit a 20% higher strength than unreinforced beams. The lower strength of beams reinforced with basalt bars may be related to the lower modulus of elasticity of the basalt itself.

## 1. Introduction

Literature on the subject concerning research, theoretical, and numerical models as well as execution of reinforcements for building structures is extensive. Reinforcement refers both to existing structures, e.g., historical buildings of architectural value, and newer and currently designed structures.

In the case of objects of historical value, reinforcement is used primarily as a preventive measure and it is introduced in valuable or historical buildings in order to extend the period of their usability or service life. Moreover, measures commonly applied in engineering practice combining reinforcement with preservation aim at the restoration of the original function to a weakened or damaged existing structure, or its modernisation and adaptation to new loads or a new function. 

In the case of new, currently designed structures, we may observe a trend towards the construction of composite structures, utilising properties of various materials, e.g., through reinforcement in the tensile zone of wooden beams produced from inferior quality timber [1,2,3,4,5,6,7,8]. Among other things, these measures aim at the optimisation of their cross-sections and increased use of inferior quality materials, in this case timber; additionally, such elements are relatively easy to manufacture. Application of a relatively low percentage share of composite materials in the section makes it possible to considerably enhance strength, load-bearing capacity, rigidity, or durability of such structural elements, as well as alter the element failure mechanism. 

Currently, it is a common practice to reinforce wooden bar structures using composite materials, such as e.g., fiber-reinforced polymers (FRP) in the form of bars, tapes, bands, mats, meshes, or strings. Reinforcements bonded to wooden elements using resins and adhesives predominate, while mechanical fasteners are less common. The most common polymer composites used in wooden structures include materials based on carbon fibers (carbon fiber-reinforced polymers—CFRP), basalt fibers (basalt fiber-reinforced polymers—BFRP), glass fiber (glass fiber-reinforced polymers—GFRP) and aramid fiber (aramid fiber-reinforced polymers—AFRP). Next to polymer materials reinforcements include also plant origin fibers [9], e.g., jute [10], linen [11], or bamboo [2].

In the case of tests and analyses conducted on beams, the aim of research is typically to determine the effect of the applied reinforcement on an increase in strength, load bearing at bending or shear strength, as well as identify the type of failure mechanism under static loads either within a short time interval or under long-term loading [12,13,14,15].

Wdowiak-Postulak and Brol [4] tested beams manufactured from glued pine timber type c (combined), of which each was composed of four lamellas. Bending reinforcement CFRP tapes were glued to the bottom face of the beam or between the two bottom lamellas. This provided a 23% increase in load-bearing capacity and a 36.29% increase in rigidity. Moreover, reinforcement had a positive effect on the structural durability of the beams. Additionally, an analytical model was proposed as a useful tool in calculations for such beams, showing high consistency with empirical testing results. 

In turn, Johns and Lacroix [16] tested beams with glued CFRP tapes in the tensile zone. An increase was recorded in the load-bearing capacity of reinforced beams, reaching as much as 70% in relation to that of non-reinforced beams. In turn, a study by [17] presented testing results for several types of beams made from solid timber, in which bending reinforcement was provided by steel plates and CFRP tapes, while shear strength was increased using self-drilling bolt fasteners. It was shown that beams with steel plates had a greater load-bearing capacity compared to beams reinforced with CFRP tapes. In the case of shear reinforcement provided by screws, the load-bearing capacity increased by as much as 44%.

Kociszewski and Gozdecki [18] presented research results for new beams manufactured from glulam reinforced with glass fiber GFRP, which was glued between lamellas in the compression and tensile zones. This effective, relatively simple, and inexpensive reinforcement method produced an increase in rigidity by 7.2%, at the theoretic increase of 8.1%.

Interesting studies on three-span beams reinforced with steel rods bonded in the tensile zones were conducted by Lukin [19], who determined the optimal degree of reinforcement to eliminate the risk of failure as a result of brittle cracking.

From the literature review and analysis of Science Direct databases (2010–2020), out of 94 articles, only two refer to the reinforcement of beams with ribbed steel and seven refer to BFRP bars. Most of the papers are related to reinforcement with smooth steel and timber beam carbon fibre. Furthermore, pine timber beams have been investigated previously [13,20,21], but graded timber was typically used. In contrast, it was decided in this study to assess the effectiveness of reinforcement with smooth and ribbed steel rods as well as BFRP composite rods in glued beams manufactured from pine timber with some defects and on this basis evaluate the applicability of this reinforcement method in industrial practice, i.e., the manufacture of prefabricated elements (beams) reinforced with composites. Since timber is a natural material, wood defects are unavoidable when sourcing lumber for glued elements. Depending on the type and intensity of occurrence, they significantly weaken the wood element in which they occur. One of the ways to increase quality, including strength of the wood, is to remove the defects and re-join the resulting pieces into one element. Such a process allows reducing the cross-section of manufactured elements/beams; however, in the case of reinforced beams, which are manufactured in predetermined specific dimensions, it may not always be required. 

## 2. Experimental Material

Analyses were conducted on pine timber, the so-called main yield and side boards. Reinforcement of the tensile zone was provided by steel and basalt rods. 

**Figure 1:**
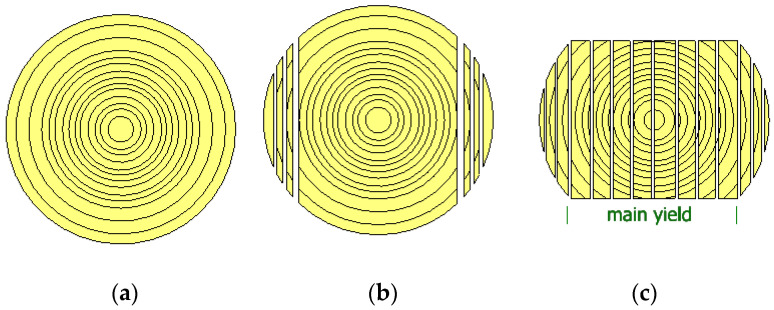
A schematic diagram of timber conversion: (**a**)—cant sawing, (**b**)—side board sawing, (**c**)—main yield.

The main yield was obtained from cant breakdown after side boards were sawn off (Figure 1). The main yield dimensions were 137 mm × 39.50 mm × 3485 mm (width × thickness × length), whereas side boards were of the same length and widths but varied in thickness from 20 to 22 mm. The main yield was obtained from sawing of round timber (stem timber) harvested in five Forest Districts, Olesno (50°52′30″ N 18°25′00″ E), Kalisz Pomorski (53°17′54″ N 15°54′21″ E), Dąbrowa Tarnowska (50°10′29″ N 20°59′11″ E), Wymiarki (51°30′44.36″ N 15°05′00.13″ E), and Biała Podlaska (52°02′ N 23°07′ E). In turn, no complete timber origin records were available for side boards. The method of stem log sawing into logs and next into sawn timber, as well as the quality of that timber were described in more detail in earlier papers [22,23].

**Figure 2:**
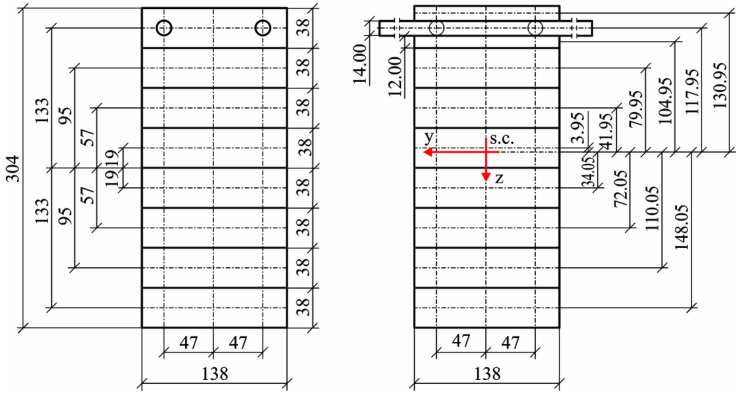
A schematic diagram of designed beams.

Prior to analyses, the main yield was trimmed on a thickness planer to a thickness of approximately 38 mm, while side boards were trimmed to a thickness of 19 mm so that both planks jointly were of the total thickness of one plank from the main yield. For each plank of the main yield, their density and modulus of elasticity were determined. The modulus of elasticity (E) was determined based on the deflection caused by a load of 382.6 N. Side boards were only tested visually, so as to ensure that no rotten knots or knot holes were located at the milling sites for the grooves’ housing rods. In each of the side boards, a recess was milled at a distance of approximately 47–48 mm from the axis (Figure 2). Groove depth was dependent on the quality of used rods, i.e.,:

– For smooth steel rods with a nominal diameter of 14 mm, grooves of 7.05 mm in radius were prepared;
– For basalt rods (coated polymer rods) of 14 mm in diameter, grooves of 7 mm in diameter were prepared;
– For ribbed steel rods of 14 mm in diameter grooves of 7.2 mm in depth and 14.6 mm in width were prepared.

Ribbed steel has longitudinal and transverse ribs of approximately 0.8 mm in height each. An additional problem with the placement of these rods in the grooves results from the spiral rib arrangement. The seam linking the ribs is not located along the rod axis but runs spirally along its length; thus, grooves for these rods needed to be deeper. In addition, smooth rods for concrete reinforcement are not perfectly round in their cross-section and show considerable deviations from the assumed nominal diameter, with the differences amounting up to approximately 0.2 mm. It was attempted to counter this imperfection to a certain extent by increasing the pressure applied in the pressing process. For this reason, for smooth steel, the pressing pressure was 0.48 MPa, i.e., close to that used for beams with no steel reinforcement elements, while in the case of coated polymer rods, in view of their quality, the pressure amounted to 0.58 MPa. In turn, in order to completely seal the beam system containing ribbed rods, the pressure of approximately 0.72 MPa was required. The basic characteristics of rods used in the tests are given in Table 1. Basalt rods exhibit an over 4-fold lower density, and simultaneously, their modulus of elasticity was over 4.5-fold lower. 

From the selected timber elements under conditions comparable to those of industrial-scale production, glued beams of 137 mm × 300 mm at the cross-section were manufactured, composed of seven layers of main yield planks and two layers of side boards. Except for the outer layers containing reinforcement rods, the selection of lamellas for beam manufacture was determined by the established value of the modulus of elasticity (MOE). Young’s modulus was assessed in a 4-point bending test.

Manufactured sets, directly before they were glued into beams, were planned to provide quality surface for gluing. Onto such prepared surfaces, melamine–urea–formaldehyde resin (MUF) 1247 was applied at 220–240 g/m^2^. The dedicated hardener 2526 was used. Both products were produced by Akzo Nobel. The mixture was prepared taking into consideration the conditions found in the laboratory facility. The hardener was added at 20 g per 100 g resin. As it was mentioned above, pressing pressure was selected depending on the type of rods to ensure complete sealing of the layers containing the rods. Due to the amount of used hardener, beams were left in the press under pressure close to the operating pressure for a minimum of 270 min. For the first 30 min, the operating pressure was controlled. 

**Figure 3:**
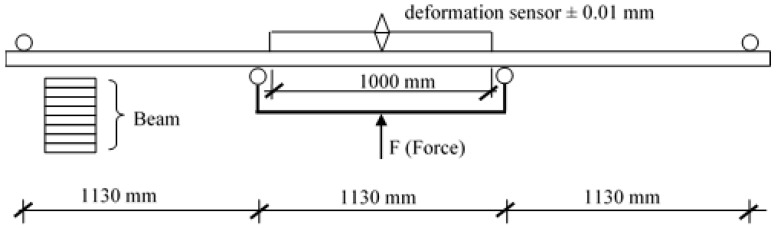
A loading scheme for glued beams.

Manufactured beams were tested to determine bending strength in a 4-point bending test following the scheme presented in Figure 3. The assessment was made in accordance with the EN 408 standard on the characterization of solid wood and cross-laminated wood [24]. The modulus of elasticity was assessed by applying a load of approximately 30 kN on each beam. A total of 10 such tests were performed, with the results recorded only for the last five tests. First, the applied load amounted to 3 kN, at which point the measurements of force and deformation were reset. Force was recorded using a dedicated recording device, while deformation was determined using a linear deformation sensor (0–50 mm, 0.01 scale). Upon the completion of measurements used to determine the modulus of elasticity, the deformation sensor was removed from the beam, and a transverse load was applied in order to determine bending strength. Linear dimensions of the cross-section were determined using a SYLVAK caliper (range 0–350 mm, 0.01 mm scale), while beam length was measured using a measuring rule by Stanley (0–5000 mm, 1 mm scale). Moisture content was recorded with a hygrometer.

Values of the modulus of elasticity for individual lamellas to specific zones of the beams were selected so as to provide a relative symmetry in relation to the beam center. 

The modulus of elasticity of the lumber was obtained in a four-point bending test as described previously [22].

Mean values of the modulus of elasticity in individual layers for the manufactured beam types are given in Table 2. As it results from the data presented in that table, the moduli of elasticity differ relatively significantly only in the outermost lamella of the compression zone. As a result of the limited availability of timber, it was impossible to produce identical systems. Twelve beams each were manufactured with the two types of steel rods, while 16 beams were made with basalt rods.

In order to determine the basic characteristics of manufactured beams, the following assumptions were made for calculations:

– The cross-section of the designed beam is 138 mm × 304 mm,
– The axis of rods is located at a depth of 19 mm from the tensile zone surface/the face,
– The orifice is an ideal circle of 14.2 m in diameter,
– The modulus of elasticity of lamellas containing rods is 11.5 kN/mm^2^,
– The modulus of elasticity of steel is 200 kN/mm^2^, while that of basalt rods is 45 kN/mm^2^.

For these assumptions, the components were determined to calculate the principal moment of inertia in relation to the y-axis. The determined values are given in Table 3. The division into 10 layers was adopted, dividing the package containing rods into three parts, with two of them manufactured from wood alone and one reinforced.

The substitute value for the modulus of elasticity of the layer with rods was adopted from the dependence (1):

(1) $$E=V_{1}\cdot E_{1}+V_{2}\cdot E_{2}$$

where

V—the volume fraction of a given phase,
E—Young’s modulus (longitudinal modulus of elasticity) of a given phase.

Thus, the substitute modulus of elasticity for the zone/layer containing steel rods in accordance with Equation (2) will be 41.5 kN/mm^2^:

(2) $$\mathrm{Ep}=\frac{\left(\frac{\pi \cdot 14^{2}}{4}\cdot 2\right)}{138\cdot 14}\cdot 200+\frac{138\cdot 14-\frac{\pi \cdot 14^{2}}{4}\cdot 2}{138\cdot 14}\cdot 11.5=41.5\;\mathrm{kN}/{\mathrm{mm}}^{2}$$

while for basalt rods, it was only 16.8 kN/mm^2^ (3):

(3) $$\mathrm{Ep}=\frac{\left(\frac{\pi \cdot 14^{2}}{4}\cdot 2\right)}{138\cdot 14}\cdot 45+\frac{138\cdot 14-\frac{\pi \cdot 14^{2}}{4}\cdot 2}{138\cdot 14}\cdot 11.5=16.8\;\mathrm{kN}/{\mathrm{mm}}^{2}.$$

In order to simplify the estimation of rigidity for the designed beams, the last three layers were replaced by one layer, and for this layer, the substitute modulus of elasticity was determined. It amounted to 22.49 kN/mm^2^ when it was reinforced with steel rods, while it was 13.44 kN/mm^2^ when it was reinforced with basalt rods. This considerable difference between the quality of the system containing steel rods and those containing basalt rods does not contradict the concept of this study. It was assumed that the proposed solution would not only cause a marked increase in rigidity of glued laminated beams but first of all limit the effect of wood defects, while additionally, it would have a positive impact on a reduction in the scatter of bending strength for these beams. 

Following the bending strength tests for selected samples, specimens of approximately 70 × 70 × 150 mm^3^ were collected in order to evaluate the quality of rod bonding using computer tomography. Scanning was performed with the use of a Hyperion X9Pro tomograph with objects scanned with the resolution of 0.3 mm at lamp voltage of 90 kV, resolution 68 m, imaging field 13 cm × 16 cm (MyRay, Via Bicocca, Imola-Bo, Italy).

The recorded results of direct measurements were analysed statistically using the Statistica13.0 package (StatSoft Inc., Tulsa, OK, USA).

## 3. Discussion and Results

**Figure 4:**
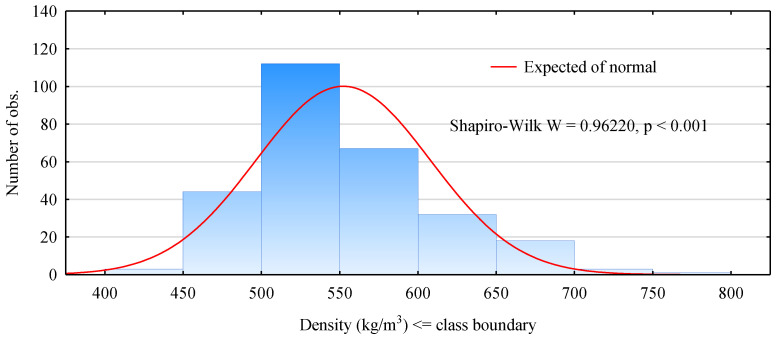
A histogram of the distribution of density for tested timber.

Although the primary aim of the study did not include qualitative evaluation of pine timber, it was decided to provide some information on the tested material. In the case of a material free from defects, there is a strong relationship between its strength, or generally its mechanical properties, and its density. However, in a material with defects, and in the case of wood as a natural material it is true for each ungraded timber piece, this dependence is disturbed. For most mechanical properties, the presence of defects reduces the value of a given characteristic compared to the defect-free material. One of the significant relationships is connected with the dependence of the modulus of elasticity on wood density. A given timber element was ascribed its position in the designed beam depending on its modulus of elasticity assessed in the bending test. Unfortunately, it is a time-consuming process and burdened with drawbacks. In contrast, density is a parameter that is relatively easy to assess. Moreover, information on density is required at the classification of timber to individual grades of structural timber. According to the PN-EN 318 standard, a given timber element is classified to a given grade not only based on an assessment of its mechanical properties but also its density. A histogram of densities of the tested timber elements is presented in Figure 4. As it results from these data, the population selected for these analyses does not exhibit a normal distribution in terms of its density. Although the range of densities is wide, i.e., from 400 to 800 kg/m^3^, planks with the range from 450 to 600 kg/m^3^ predominate.

**Figure 5:**
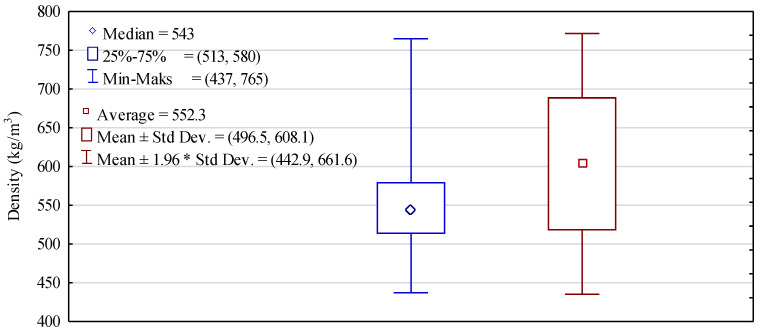
A box plot for statistical data concerning density of tested timber.

The use of the sample mean as a measure of the central trend is common, and typically, it rather well describes the sample median. Unfortunately, it is related to a normal distribution. Figure 5 presents box plots for the assessment of density based on the mean and median. The mean density of timber differs by as little as slightly below 10 kg/m^3^, i.e., less than 2% from the median. Half of the tested population is characterised by densities between 513 and 580 kg/m^3^; i.e., it is high but consistent with other data concerning density of pine originating from Poland. However, it needs to be stressed that even timber of the lowest density meets in this respect the requirements of the standards for structural timber of high mechanical parameters.

**Figure 6:**
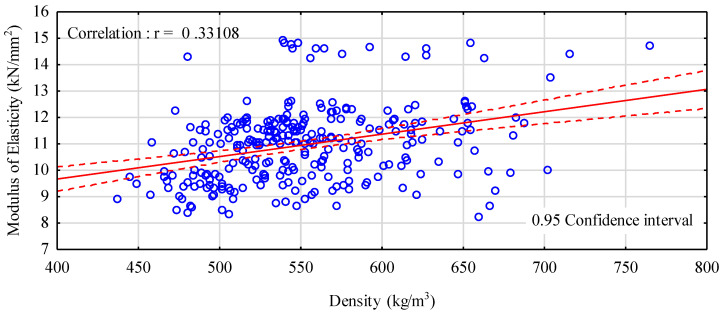
A correlation between density and the modulus of elasticity determined in a four-point bending test.

Figure 6 presents a dependence between density and the modulus of elasticity. This correlation is definitely very low. A certain trend is observed—as it had been expected, the modulus of elasticity was growing with an increase in density, although this increase was not markedly evident. It needs to be stated here that irrespective of density, there are planks with a modulus of elasticity greater than 13 kN/mm^2^. They are probably planks with very few defects or even defect-free.

**Figure 7:**
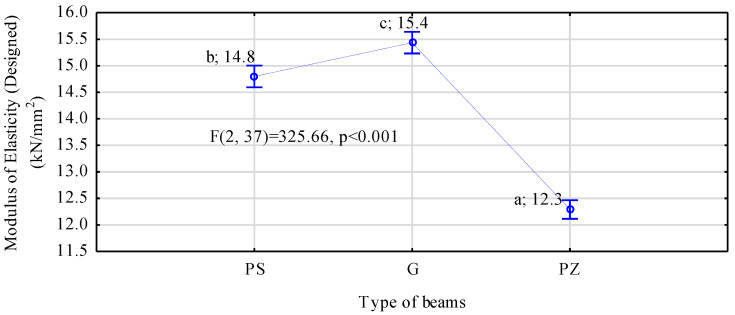
ANOVA of the designed modulus of elasticity of beams reinforced with rods (a, b, c letters mark homogenous groups in the HSD Tukey test).

As mentioned earlier, when beams were being manufactured, no wood defects were removed, and timber was selected based solely on its modulus of elasticity. Timber was selected so that the highest possible number of planks with a low modulus of elasticity, i.e., below 9 kN/mm^2^, could be obtained in the main yield, and only missing elements were supplemented with timber with higher modulus values. Due to the limited availability of timber, it was impossible to design beams reinforced in the tensile zone to have an identical modulus of elasticity (Figure 7). Beams containing ribbed steel rods are characterised by a slightly lower modulus of elasticity compared to beams reinforced with smooth steel. A lower modulus of elasticity in beams manufactured with reinforcing basalt rods results from the lower modulus of elasticity of these rods, since in the beam structure, the quality of used timber was very similar, and the differences did not exceed several percent (5.5%). If basalt rod had been replaced with ribbed rods, such designed beams would have been characterised by the designed modulus of elasticity amounting to approximately 15 kN/mm^2^.

**Figure 8:**
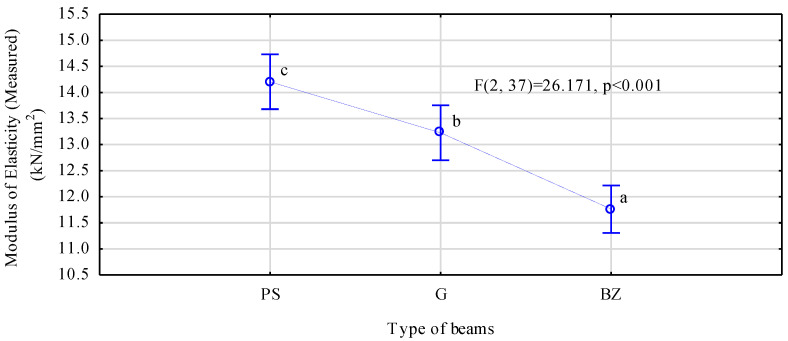
ANOVA for the modulus of elasticity of beams reinforced with rods determined in the bending test (a, b, c letters mark homogenous groups in the HSD Tukey test).

Mean values of the moduli previously determined in the bending test differ statistically (Figure 8). However, in this case, the highest modulus was recorded for beams manufactured using ribbed rod reinforcement, while it was lower for beams with smooth steel rods, which was the opposite trend to that for the designed beams. In both cases, beams manufactured using basalt rods have the lowest moduli of elasticity. The modulus of elasticity for beams reinforced with ribbed steel is approximately 7.5% higher than that of beams with smooth steel reinforcement.

**Figure 9:**
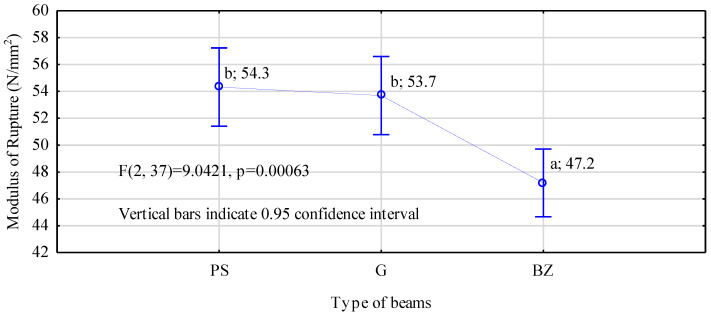
ANOVA for bending strength of rod-reinforced beams (a, b letters mark homogenous groups in the HSD Tukey test).

In turn, it has to be stressed here that all the manufactured beams are characterized by a lower moduli of elasticity than the assumed values. In the case of beams containing basalt rods and ribbed steel rods, the differences were relatively small and did not exceed 5%. In contrast, for beams containing smooth steel rods, this difference was as high as 15%. This may have been a result of several causes. Thus, it may have been caused by an inferior fastening and a lack of mechanical anchoring in the rod–timber contact zone, as well as the substandard quality of grooves prepared for the rods or their excessively large diameter in relation to the rod core. Another cause may have been the quality of the side boards used in the tests, which may have been lower than it had been assumed, since it was assessed only visually. However, the mechanical strength of beams containing steel rods, irrespective of their types, was almost identical and amounted to approximately 54 N/mm^2^ (Figure 9). These are values over 35% higher than those obtained for beams of a comparable cross-section area manufactured on the same workstations and described in a paper by Mirski et al. [22]. Thus, it is a rather significant increase in value, since in the case of beams containing basalt rods, the maximum increase in the value is 20%. 

**Figure 10:**
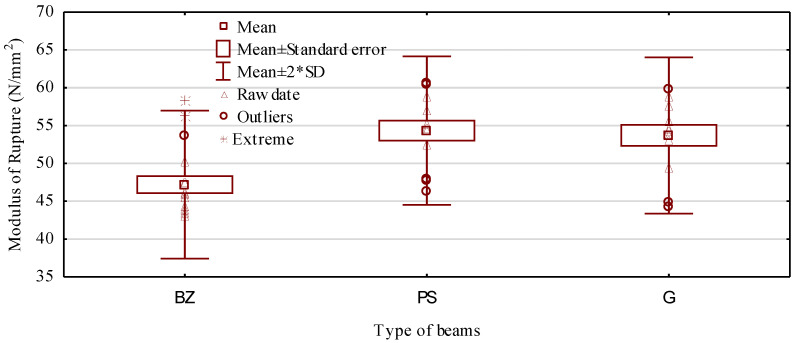
Characteristic values of static bending strength for manufactured beams.

In turn, the characteristic value, i.e., the value of the 5th percentile for the manufactured beams was 46.3 N/mm^2^, 44.3 N/mm^2^, and 42.9 N/mm^2^, respectively, for beams containing ribbed, smooth, and basalt rods (minimum value in Figure 10). Thus, the differences between individual types of beams are not very large and do not exceed 8%. However, reinforcement of the tensile zone did not provide the static bending strength of the manufactured beams fluctuating around a certain assumed value. Thus, no beams with extremely different strength values will be produced. Differences between the maximum and minimum values are smaller than those recorded in earlier studies; however, they are not as marked as it was assumed. At present, they are on average around 15.0 N/mm^2^, while for non-reinforced beams, this range was 18.9 N/mm^2^ [22].

**Figure 11:**
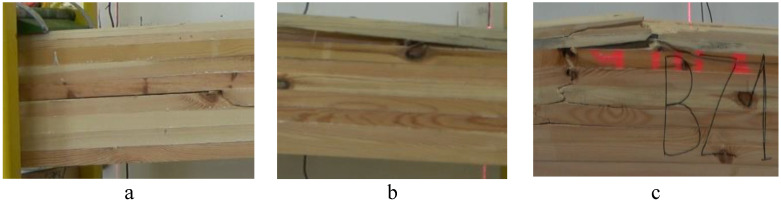
Examples of damage in designed beams (1:15 scale): (**a**) lamella 4/5 (PS), (**b**) knot in lamella 7 (G), (**c**) timber in the tensile zone (example BZ beam no. 1).

Table 4 presents probable causes for failure in the designed beams, while Figure 11 shows examples of observed damage. As it results from the presented information, in the case of beams manufactured using ribbed rods, the most common damage was caused by delaminating forces, although it was only slightly more frequent than the other types of damage. Delaminating forces cause failure of the glue line or the wood-adhesive structure in the beam core. Thus, it may be assumed that the combination of ribbed rods with timber is strong enough for the obtained layer/lamella to transfer tensile forces, whereas shearing stresses (delaminating stresses) amounting on average to approximately 3.1 N/mm^2^ start to cause beam failure. This is a rather surprising finding, since the static resistance of glue lines to splitting is approximately 7 N/mm^2^, while the shear strength of pine wood is approximately 10 N/mm^2^. As a consequence, nothing seemed to indicate failure in that zone. Beams reinforced with smooth rods fail in a different manner than it is observed for beams reinforced with ribbed rods. In this case, beam failure is determined mainly by wood defects in the tensile zone. They are knots and twisting caused by the presence of knots located directly underneath the steel-reinforced layer. Since the mean strength of both beam types is comparable, it seems to indicate the possibility of the produced glue line being inferior in quality when they were being manufactured. The expected type of beam failure was observed only during strength testing of beams reinforced with basalt rods. In this case, failure was found in the timber, in which rods were directly glued. 

**Figure 12:**
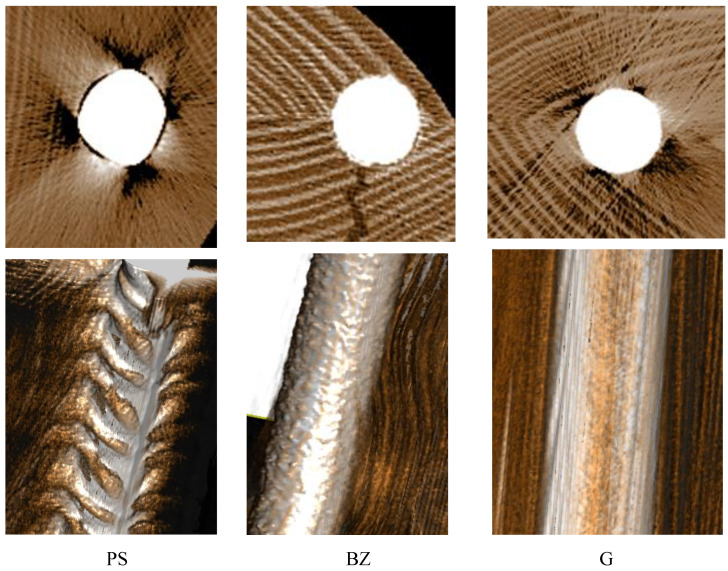
CT scans (1:1 scale). **Upper** image line—crosswise view, **lower** image line—longitudinal view.

A significant factor potentially affecting the modulus of rupture of manufactured beams is connected with the adequate bonding of reinforcement with the adjacent timber. Since rods do not have an ideally round cross-section and practically it is impossible to ensure an ideal diameter of grooves milled in timber, many factors are involved when attempting to ensure good contact of these elements in such a situation. In this case, the deviations in diameters and groove quality may be compensated for by the thickness of the glue line as well as the volume of clamping pressure for the bonded layers. Figure 12 presents tomograph scans. Although a combination of wood and steel is relatively difficult to present by tomography, particularly when steel takes a considerable section of the scanned object, the image of the wood–steel system is satisfactorily presented. These scans show a better attachment of basalt rods and smooth steel rods to the prepared grooves. When analyzing samples containing ribbed rods, it may be observed that solely the rod ribs, and even then it is only partly, are pressed to wood. Since for smooth and ribbed rods, a similar range of strength values was obtained, it may be expected that a smaller area of the effective glue line at the application of ribbed rods is compensated for by the ribs being pressed into the wood structure. 

As it results from the data presented in Table 4, beams manufactured using smooth rods fail more frequently in the tensile zone when compared to the beams containing ribbed rods. Since mean bending strength in comparable in both beam types, this means that ribbed rods are more firmly anchored in the timber in comparison to smooth rods. The indentations in wood observed on the scans may enhance anchoring of the steel in wood. This seems to be confirmed by the fact that the modulus of elasticity of PS beams exceeds greatly that of G beams (Figure 8). The designed modulus of elasticity of the G beams was higher than that of the PS beams; thus, at an identical anchoring, this should be similar also after the measurement. In view of the above, it may be concluded that the performed scans on the one hand confirm a positive effect of ribbing, while on the other hand, they indicate a certain potential for the further pressing of ribbed steel into the wood.

## 4. Conclusions

The potential to increase rigidity as well as load-bearing capacity of beams manufactured from glued laminated timber (glulam) thanks to the bonding of an additional element with a markedly higher modulus of elasticity than wood has been successfully used for years. However, so far, this procedure either improved the load bearing of already existing structures, or the rigidity was increased in very long, newly designed elements. In the former case, typically, only the tensile zone was reinforced, while in the latter case, usually, symmetric beams were manufactured. Moreover, while in the former case, the initial quality of structural timber was unknown, in the latter case, graded timber was selected, and major defects were eliminated. On the basis of the conducted tests, it seems that reinforcement may be applied also to meet another objective, i.e., to more efficiently utilise inferior quality timber. In this case, reinforcement provides a higher strength of the beams, even when using low-quality timber. Specific conclusions from the performed tests and analyses include the following:

– An increase in static bending strength of beams amounts to approximately 120% strength of non-reinforced beams,
– The rigidity of manufactured beams assessed in the 4-point bending test is comparable to the calculated rigidity; the differences do not exceed several percent,
– The strength of beams reinforced with steel is by approximately 15% higher than in the case of basalt rod reinforcement; however, the increase in strength is consistent with the increase in rigidity of steel-reinforced beams,
– A lack of uniformity in the cross-sections of ribbed rods, and the resulting inferior contact of the timber–adhesive–rod system is probably compensated for by the pressing of the ribs into the wood structure and the capacity for mechanical meshing of rod ribs pressed into wood,
– The expected reduction in the scatter of bending strength values was not obtained in such manufactured beams.

## Figures and Tables

**Table 1:** Physical and mechanical properties of tested rods.

Property/Rod	Smooth Steel Rod	Ribbed Steel Rod	Basalt Rod
Nominal diameter (cm)	14	14	14
Rod diameter (cm)	14	13	13.6
Type	A-0, St0S	A-II 18G2	BFRP
R_e min_ (MPa) *	195	355	-
R_m_ (MPa) **	315	420	850
E (GPa) ***	210	210	45
Density (g/cm^3)^	7.85	7.85	2.0
Electric conductivity	good	good	Non-conducting material
Resistance to corrosion and alkaline environment:	No resistance to corrosion	No resistance to corrosion	Very high

* tensile strength at yield point. ** tensile strength. *** longitudinal elasticity modulus.

**Table 2:** Mean moduli of elasticity of lamellas used to manufacture beams.

Property	Number of Lamellas—Counting from the Compression Zone						
	7	6	5	4	3	2	1
**Beams with steel rods—ribbed steel—(PS)**							
E (kN/mm^2^)	11.78	10.63	9.10	9.13	10.67	11.75	12.89
SD (kN/mm^2^)	0.22	0.49	0.32	0.31	0.55	0.17	2.11
**Beams with steel rods—smooth rods—(G)**							
E (kN/mm^2^)	11.61	10.56	9.19	9.21	10.54	11.60	14.50
SD (kN/mm^2^)	0.20	0.55	0.46	0.46	0.56	0.20	0.22
**Beams with basalt rods—(BZ)**							
E (kN/mm^2^)	11.73	10.57	9.76	9.99	10.78	11.69	12.45
SD (kN/mm^2^)	0.33	0.49	0.42	0.59	0.26	0.32	0.11

**Table 3:** Components of the principal moment of inertia in relation to the y-axis.

Lamella	B × h	I	A	d	A·d^2^	I_w_	E_i_
	mm·mm	cm^4^	cm^2^	cm	cm^4^	cm^4^	N/mm^2^
1	138·38	63.10	52.44	13.3	9276	9339	-
2	138·38	63.10	52.44	9.5	474	4796	-
3	138·38	63.10	52.44	5.7	1704	1767	-
4	138·38	63.10	52.44	1.9	189	252	-
5	138·38	63.10	52.44	1.9	189	252	-
6	138·38	63.10	52.44	5.7	1704	1767	-
7	138·38	63.10	52.44	9.5	4733	4796	-
8	138·12	1.987	16.56	12	2385	2387	11,500
9	138·14	3.156	19.32	13.3	3418	3421	p
10	138·12	1.987	16.56	14.6	3530	3532	11,500
total	-	-	419.5	-	-	-	-

**Table 4:** A list of probable causes of failure in designed beams (number of the lamella counting from the compression zone).

Beam No.	Beam Type		
	PS	G	BZ
1	lamella 5/6 ^1^	knot in lamella 7	timber in the tensile zone
2	lamella 4/5	knot in lamella 7	timber in the tensile zone
3	knot in lamella 7	timber in the tensile zone ^2^	knot in lamella 7
4	knot in lamella 7	knot in lamella 7	timber in the tensile zone
5	lamella 4/5	timber in the tensile zone	timber in the tensile zone
6	lamella 5/6/4	no identification	knot in lamella 7
7	timber in the tensile zone	knot in lamella 6	timber in the tensile zone
8	knot in lamella 7	no identification	timber in the tensile zone
9	knot in lamella 6	timber in the tensile zone	timber in the tensile zone
10	lamella 4/5	timber in the tensile zone / knot in lamella 7	timber in the tensile zone
11	lamella 5/6	timber in the tensile zone	knot in lamella 7
12	knot in lamella 7	knot in lamella 5	timber in the tensile zone
13	-	-	timber in the tensile zone
14	-	-	shear in glue line 3
15	-	-	knot in lamella 7
16	-	-	timber in the tensile zone

^1^—shear in glue line between successive lamellas, ^2^—damage to side board containing glued reinforcement (rods).

## Data Availability

The data presented in this study are available on request from the corresponding author.
